# Selective inference for fMRI cluster-wise analysis, issues, and recommendations for critical vector selection: A comment on Blain et al.

**DOI:** 10.1162/imag_a_00198

**Published:** 2024-06-24

**Authors:** Angela Andreella, Anna Vesely, Wouter Weeda, Jelle Goeman

**Affiliations:** Department of Economics, Ca’ Foscari University of Venice, Venice, Italy; Department of Statistical Sciences, University of Bologna, Bologna, Italy; Department of Psychology, Leiden University, Leiden, The Netherlands; Department of Biomedical Data Sciences, Leiden University Medical Center, Leiden, The Netherlands

**Keywords:** fMRI cluster analysis, brain mapping, multiple testing, permutation test, selective inference, true discovery proportion

## Abstract

Two permutation-based methods for simultaneous inference on the proportion of active voxels in cluster-wise brain imaging analysis have recently been published: Notip and pARI. Both rely on the definition of a critical vector of orderedp-values, chosen from a family of candidate vectors, but differ in how the family is defined: computed from randomization of external data for Notip and determined a priori for pARI. These procedures were compared to other proposals in the literature, but an extensive comparison between the two methods is missing due to their parallel publication. We provide such a comparison and find that pARI outperforms Notip if both methods are applied under their recommended settings. However, each method carries different advantages and drawbacks.

## 1 Introduction

Cluster-extent-based thresholding is a common approach in functional Magnetic Resonance Imaging (fMRI) analysis to explore which parts of the human brain are activated under some stimuli of interest. This approach permits controlling the Type I error at the level of clusters of adjacent voxels, gaining power with respect to voxel-wise inference approaches by exploiting the intrinsic spatial structure of fMRI data (Nichols & Hayasaka, 2003).

However, the method is affected by the so-called spatial specificity paradox. This paradox arises because the larger the identified cluster, the less information we obtain from classic cluster inference about the signal within it. Indeed, the method tests the null hypothesis that none of the voxels in the cluster are active. Rejecting this null hypothesis only allows to claim the presence of at least one active voxel within the cluster. Consequently, larger clusters provide less information about the number and spatial location of active voxels (Woo et al., 2014). Moreover, conducting follow-up inference within the cluster, or “drilling down,” introduces a “double-dipping” problem and leads to an inflated Type I error rate (Kriegeskorte et al., 2009).

The spatial specificity paradox can be resolved by making post-hoc inference on the True Discovery Proportion (TDP), that is, the proportion of false null hypotheses within a subset. In neuroimaging, post-hoc TDP inference procedures provide lower confidence bounds on the proportion of active voxels within clusters, simultaneously over all possible clusters of interest. The simultaneity characteristic of the confidence bounds makes them valid even under post-hoc selection, allowing for follow-up inference within the cluster, unlike the cluster-extent-based thresholding approach (Goeman et al., 2023;Rosenblatt et al., 2018).

The first approach that proposed simultaneous inference on TDP in the fMRI context is the “All-Resolution Inference” (ARI) method developed byRosenblatt et al. (2018). However, ARI is parametric and can have low power in some scenarios, especially if correlated data such as fMRI are analyzed. It is well known that statistical analyses based on the permutation theory are superior in terms of power and underlying assumptions in fMRI data analysis since they adapt to the correlation structure of thep-values (Helwig, 2019;Winkler et al., 2014). Permutation-based approaches to compute lower bounds for the TDP were first proposed byMeinshausen (2006)andHemerik et al. (2019). However, these methods analyze only clusters consisting of the smallestkp-values. The SansSouci method ofBlanchard et al. (2020)extended this type of permutation-based simultaneous confidence bounds for the TDP to have the same flexibility as ARI, that is, for clusters defined in different ways, even post-hoc, as many times as the researcher wants. An alternative permutation-based TDP method was proposed byVesely et al. (2023).

Two recent approaches have appeared in the literature to compute a lower bound for the TDP: Notip byBlain et al. (2022)and pARI byAndreella et al. (2023). Both methods build upon the work ofBlanchard et al. (2020), each proposing a different specific permutation-based TDP approach tailored to neuroimaging applications. In the work byBlain et al. (2022), the authors compare their methods with ARI and SansSouci; the gain in power and reliability of permutation-based approaches over parametric methods is apparent. However, due to the parallel publication process, Notip and pARI have not yet been compared to each other.Blain et al. (2022)have made a comparison with pARI, but the settings of the method used in the study were not those recommended byAndreella et al. (2023). Therefore, a proper comparative analysis is still lacking. In this manuscript, we provide such an analysis.

The paper is organized as follows.Section 2briefly revisits inference on the TDP. Subsection 2.1 gives a general formulation of the permutation methods cited above (i.e., SansSouci, pARI, and Notip) before describing in detail the similarities and dissimilarities between Notip and pARI in Subsection 2.2. Finally,Section 3revisits the analyses presented inBlain et al. (2022), comparing them to pARI as defined inAndreella et al. (2023). In this comparison, we followBlain et al. (2022)exactly in terms of the choice of the datasets and evaluation criteria. We show that we replicate the results shown inBlain et al. (2022)regarding Notip, then add the pARI method under the specifications recommended byAndreella et al. (2023). By following exactly the analysis choices made in the Notip paper, we make sure not to favor the pARI method, with which we are more familiar.

## 2 Controlling True Discovery Proportions

Consider the brainB={1,…,m}⊂ℕcomposed ofmvoxels and, for each voxeli∈B, ap-value$p_{i}$corresponding to the null hypothesis that it is not active under the condition of interest. We define byA⊆Bthe unknown set of truly active voxels and byS⊆Ba generic non-empty subset of hypotheses of interest (i.e., a cluster of voxels). For any choice ofS, interest lies in the number of true discoveriesa(S)=|A∩S|or, equivalently, the TDP|A∩S|/|S|, where|S|stands for the cardinality of the setS. For a chosen error rateα∈(0,1), TDP procedures aim to construct lower(1−α)-confidence bounds for these quantities, simultaneously over all possible choices ofS. The confidence bounds for the number of true discoveries, denoted by$\bar{a}\left(S\right)$, are such that

$$\text{Pr}\left(\bar{a}\left(S\right)\leq a\left(S\right)\right)\geq 1-\alpha$$ (1)

for allS⊆B. An analogous formulation holds for the confidence bounds for the TDP, which can be immediately derived from$\bar{a}\left(S\right)$(Goeman & Solari, 2011).

The simultaneity of the confidence bounds makes them valid even under post-hoc selection and so allows the user to decide which sets of hypothesesSto analyze in a flexible and post-hoc manner. Therefore, methods with this property give information on the amount of true signal inside any set of voxels. The collection of voxels can be defined in various ways, allowing researchers to choose the method that suits their needs. Examples include clusters based on a searchlight, anatomical regions of interest (ROIs), functional ROIs, and data-driven regions (e.g., cluster-extent-based thresholding). Users can drill down into a region multiple times to more precisely identify the location of true active voxels by applying any region selection rule, whether data-driven or not.

### 2.1 TDP based on critical vectors and permutations

To bound the TDP, pARI and Notip, like ARI and SansSouci, use a strategy based on critical vectors for orderedp-values. They compute the simultaneous lower(1−α)-confidence bound for the number of true discoveries in a clusterSas

$$\bar{a}\left(S\right)=\underset{1\;\;\leq \;\;u\;\;\leq \;\;|S|}{\text{max}}1-u+\left|\left\{i\in S:p_{i}\leq \ell_{u}\right\}\right|$$ (2)

where$\ell =\left(\ell_{1},\ldots ,\ell_{m}\right)\in {\left[0,1\right]}^{m}$is a suitable non-decreasing vector called critical vector, or in some cases template (Blain et al., 2022;Blanchard et al., 2020). Different critical vectors have been proposed, but in order to obtain valid simultaneous confidence bounds as inEquation (1), it must satisfy the following condition:

$$\text{Pr}\left(\overset{\left|N\right|}{\underset{i=1}{\cap }}\left\{q_{\left(i\right)}\geq \ell_{i}\right\}\right)\geq 1-\alpha ,$$ (3)

whereN=B \Ais the unknown set of inactive voxels, and$q_{\left(1\right)}\leq \ldots \leq q_{\left(\left|N\right|\right)}$are their sortedp-values. This means that the curve of the sortedp-values corresponding to inactive voxels should lie completely above the critical vector with probability at least1−α.

InFigure 1, we give a graphical intuition of the computation of$\bar{a}\left(S\right)$, as defined inEquation (2). The solid black line is the curve of the sortedp-values in the clusterSof interest; the dashed red and dotted blue lines are two critical vectors (of pARI and Notip, respectively). If there were no signal inS, the black curve would be completely to the left of (i.e., above) each critical vector with probability1−α. As it happens, the curve is way to the right of (i.e., below) the critical vector, indicating the presence of much signal. The lower bound$\bar{a}\left(S\right)$to the number of active voxels, according to (2), is given as the maximal horizontal distance between the curve and the critical vector. It is clear from the figure that the shape of the critical vector is crucial and that different critical vectors may give very different TDP values.

**Fig. 1.**
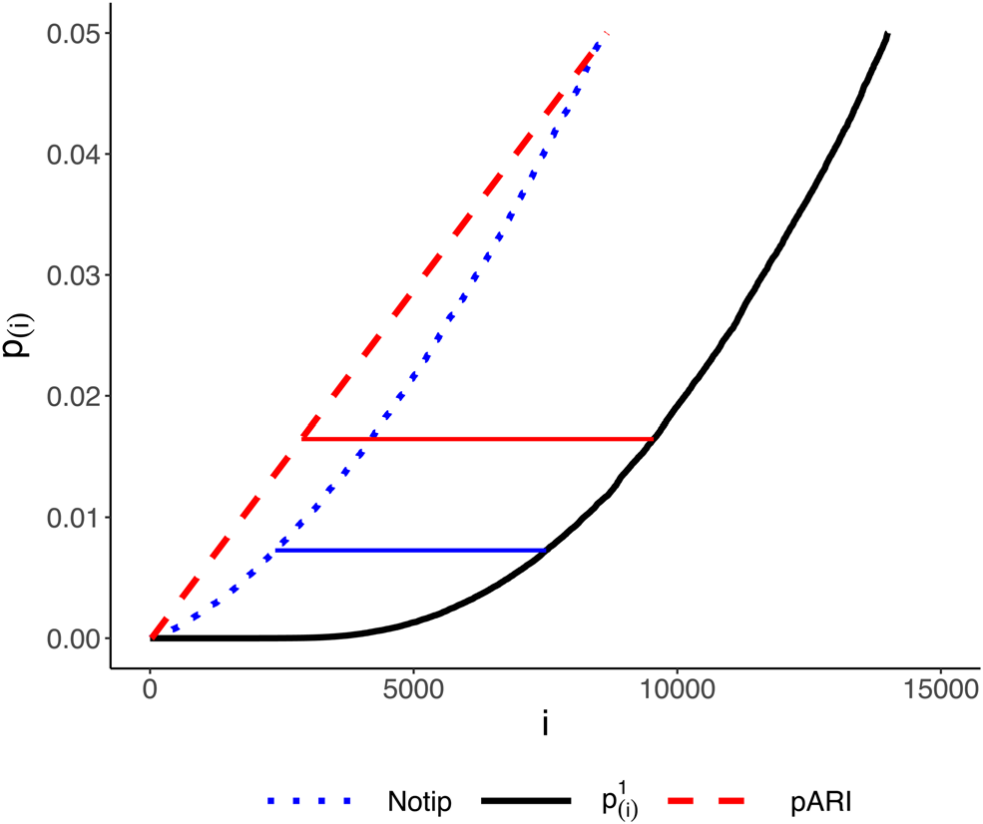
Graphical intuition ofEquation (2). The black solid line represents the vector of sorted observedp-values$p_{\left(1\right)}\leq \ldots \leq p_{\left(m\right)}$. For each method (red for pARI, blue for Notip), the broken line represents the resulting critical vector; then,$\bar{a}\left(\text{S}\right)$is computed as the length of the solid segment, which is the largest distance between the curve of the observedp-values and the critical vector.

To construct a critical vector that satisfiesEquation (3), both Notip and pARI rely on a high numberwof transformations of the data,w−1of which can be random permutations or sign-flipping transformations or any other random data transformations that preserve the distribution of the test statistics under the null hypothesis (Winkler et al., 2014), while the remaining one must be the original, untransformed data (Hemerik & Goeman, 2018). Thep-value curves arising fromw=40such data transformations are illustrated inFigure 2, with each thin grey curve ap-value curve for a permutation. To find the critical vector, a pre-specified set of candidate critical vectors$\ell \left(\lambda \right)=\left(\ell_{1}\left(\lambda \right),\ldots ,\ell_{m}\left(\lambda \right)\right)$,λ∈Λ, is chosen, such that each$\ell_{i}$is non-decreasing inλ. These candidate critical vectors are illustrated as the dashed red lines inFigure 2. In order to satisfyEquation (3), the final critical vector is chosen as the highest curve such that(1−α)100%of the sortedp-value curves lie above it. That is, if$p_{\left(1\right)}^{j}\leq \ldots \leq p_{\left(m\right)}^{j}$are the sortedp-values obtained for thej-th random permutation, thenλis chosen as the largest value such that

**Fig. 2.**
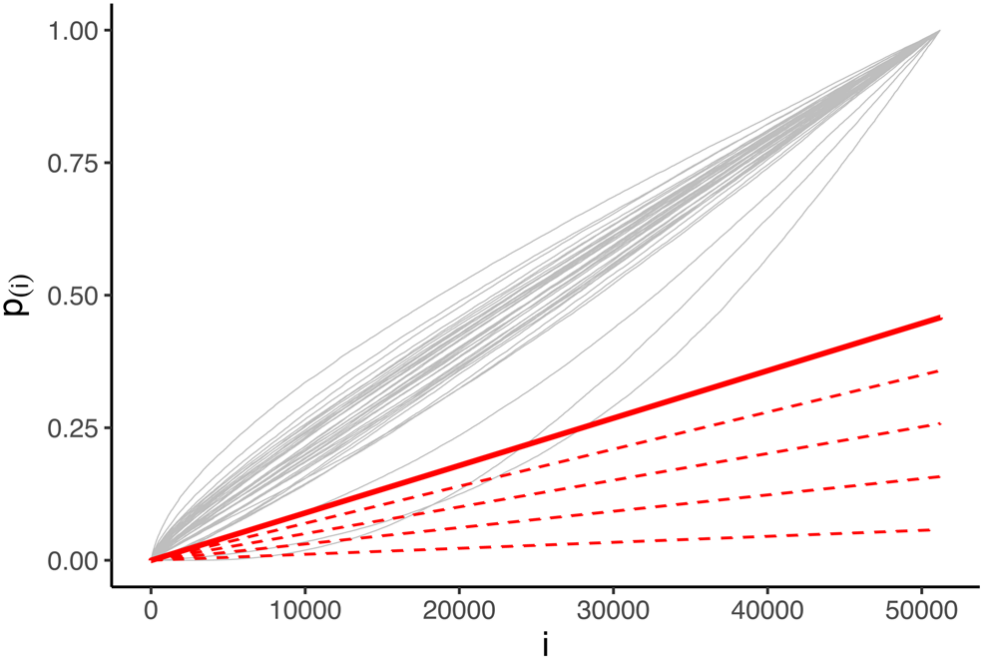
λ-calibration step: the grey lines represent the vector of sortedp-values given by a random permutation of the data randomly sampling40permutations. The red dashed lines are the candidate critical vectors for pARI having differentλvalues. The solid red line is the optimal pARI critical vector having the largestλacross the ones that cross the null distribution of thep-values represented by the grey lines at mostα%of the times.

$$|\left\{j:p_{\left(1\right)}^{j}>\ell_{1}\left(\lambda \right),\ldots ,p_{\left(m\right)}^{j}>\ell_{1}\left(\lambda \right)\right\}|\;\;\geq \left(1-\alpha \right)w.$$ (4)

The resulting critical curve is given as the thick red line inFigure 2.

This permutation-based process allows the method to incorporate the unknown spatial correlation structure of voxels in the calibration of the critical vector, and so to gain power compared to parametric methods.

### 2.2 Differences between pARI and Notip

The construction just described is common to pARI and Notip. However, pARI and Notip differ in their definition of the set of candidate vectors from which the optimal critical vector is selected, which we call a family of critical vectors (also called, in some cases, a set of learned templates as inBlain et al. (2022)andBlanchard et al. (2020)).

For neuroimaging data,Andreella et al. (2023)recommend the shifted Simes family, given by

$$\ell_{i}\left(\lambda \right)=\frac{\left(i-\delta \right)\lambda }{m-\delta }$$ (5)

whereδ∈{0,1,…,m−1}, a shift parameter, is a fixed value that must be chosen independently of the data. The SansSouci approach used the same Simes-based family defined inEquation (5)withδ=0. Choosingδlarger has the result of losing all power for clustersSof sizeδor less, but in a trade-off, this results in substantially higher power for larger clusters.Andreella et al. (2023), therefore, recommendedδ>0in general, followingHemerik et al. (2019), and substantially larger than1if interest is in large clusters. However,δis not allowed to depend on the sizes of clusters found, so a sensible default must be fixed. They recommended$\delta =3^{3}=27$when interest is on clusters of large size, as is common in neuroimaging, so we take this as pARI’s default value.

Blain et al. (2022), in contrast, define the family using$\tilde{w}$permutations on external data with$\tilde{m}\approx m$voxels. Let${\tilde{p}}_{\left(1\right)}^{j}\leq \ldots \leq {\tilde{p}}_{\left(\tilde{m}\right)}^{j}$be the sorted vector ofp-values for thej-th permutation of the external data. In the family of candidate critical vectors proposed byBlain et al. (2022),$\ell_{i}\left(\lambda \right)$is theλ-quantile of the vector$\left({\tilde{p}}_{\left(i\right)}^{1},\ldots ,{\tilde{p}}_{\left(i\right)}^{\tilde{w}}\right)$if$i\leq k_{max}$, and$\ell_{i}\left(\lambda \right)=1$otherwise, where$k_{\text{max}}\in \left\{1,\ldots ,m\right\}$is some fixed bound chosen a priori. Formally,

$$\ell_{i}\left(\lambda \right)=\{\begin{array}{cc} {\tilde{p}}_{\left(i\right)}^{\left(\left\lfloor \lambda \tilde{w}\right\rfloor \right)} & i\leq k_{max}\\ 1 & \text{otherwise,} \end{array}$$ (6)

where${\tilde{p}}_{\left(i\right)}^{\left(j\right)}$denotes thej-th smallest value among${\tilde{p}}_{\left(i\right)}^{1},\ldots ,{\tilde{p}}_{\left(i\right)}^{\tilde{w}}$.

Though seemingly similar in their use of permuted data,Equation (6)is markedly different from (4) above since (6) uses only the marginal distribution of the orderedp-values, whereas (4) uses their joint distribution. The relationship between the external data and the data under analysis should, therefore, not be seen as the usual relationship between a training and a validation set. In fact,Meinshausen (2006)proposed using the same data in (4) and (6), and thoughHemerik et al. (2019)andBlanchard et al. (2020)pointed out that doing so destroys the formal validity of the method, the choice ofMeinshausen (2006)is generally fine in practice.

In Notip,$k_{\text{max}}$is a tuning parameter, compable toδin pARI, and likeδ>0, use of$k_{max}<m$was recommended for a different family byHemerik et al. (2019). Effectively, allp-values higher than the$k_{max}$-th one are ignored by Notip. Likeδ, the choice of$k_{\text{max}}$induces a trade-off: small values can lead to a less conservative family of critical vectors but also to smaller lower bounds for the TDP.Blain et al. (2022)describe$k_{\text{max}}$as the largest size of the cluster for which a high proportion of active voxels is guaranteed. They suggested to fix$k_{\text{max}}=1,000$.

As a further improvement,Andreella et al. (2023)proposed a step-down version of pARI, which outperforms the SansSouci method in terms of power even if the same critical vector family is used. This improvement comes at the price, however, of high computational time. In this paper, we use the faster version of pARI without the step-down.

## 3 Comparison on Neurovault Data

In this section, we compare the Notip and pARI approaches, following exactly the analysis performed originally byBlain et al. (2022). The comparison between pARI and Notip methods primarily emphasizes power, as error control has been previously established in the respective papers (Andreella et al., 2023;Blain et al., 2022). The Neurovault database (Varoquaux et al., 2018) contains data from many fMRI studies. Here, we analyzed collection1952(http://neurovault.org/collections/1952), consisting of statistical maps from20different studies. First, the images were preprocessed following the procedure outlined inVaroquaux et al. (2018)(i.e., spatial normalization to MNI space using SPM12 software, resampled to a 3 mm isotropic resolution). Then, the data were preprocessed using the Python code made available byBlain et al. (2022)athttps://github.com/alexblnn/Notip, resulting in36contrast pairs. Specifically, we analyzed elementary “versus baseline,” and control contrasts from collection1952, containing data from a large number of different cognitive tasks (e.g., visual, auditory). For a complete overview of the contrasts analyzed, please refer to Table 6 inBlain et al. (2022).

The analysis was carried out using the pARI R package (https://CRAN.R-project.org/package=pARI) for applying pARI, and the Python code made available byBlain et al. (2022)for applying Notip.Figures 1and2, above, have been computed using the first dataset of this collection, that is, “shapes versus baseline” contrast versus “faces versus baseline” contrast from the HCP study. To makeFigure 1clearer, we considered the cluster composed of the smallest15,000voxels.

Here, we redo only those analyses fromBlain et al. (2022)in which they compare performance between the Notip and competing methods. It is not straightforward to compare different TDP methods because each method gives$2^{m}$TDP confidence bounds. A method that performs better for some TDP bounds may be worse for other bounds, even within the same data or simulation scenario. We followBlain et al. (2022)in their choice of metric for comparing methods, which focuses on the size of the largest cluster found at a fixed TDP threshold. Other metrics are possible; for example,Andreella et al. (2023)used the TDP of clusters defined at a fixed cluster-defining threshold as their metric. In all the analysis, we fix the number of permutations used to compute the Notip critical vector$\tilde{w}$to10,000, and the number of permutations used to calculate the null distribution of thep-values to1,000.

The left-hand side ofFigure 3reproduces the results ofBlain et al. (2022;Figure 4, right-hand side), in which they compare Notip to pARI withδ=0, that is, to SansSouci. The relative number of detections between Notip and pARI, defined as

**Fig. 3.**
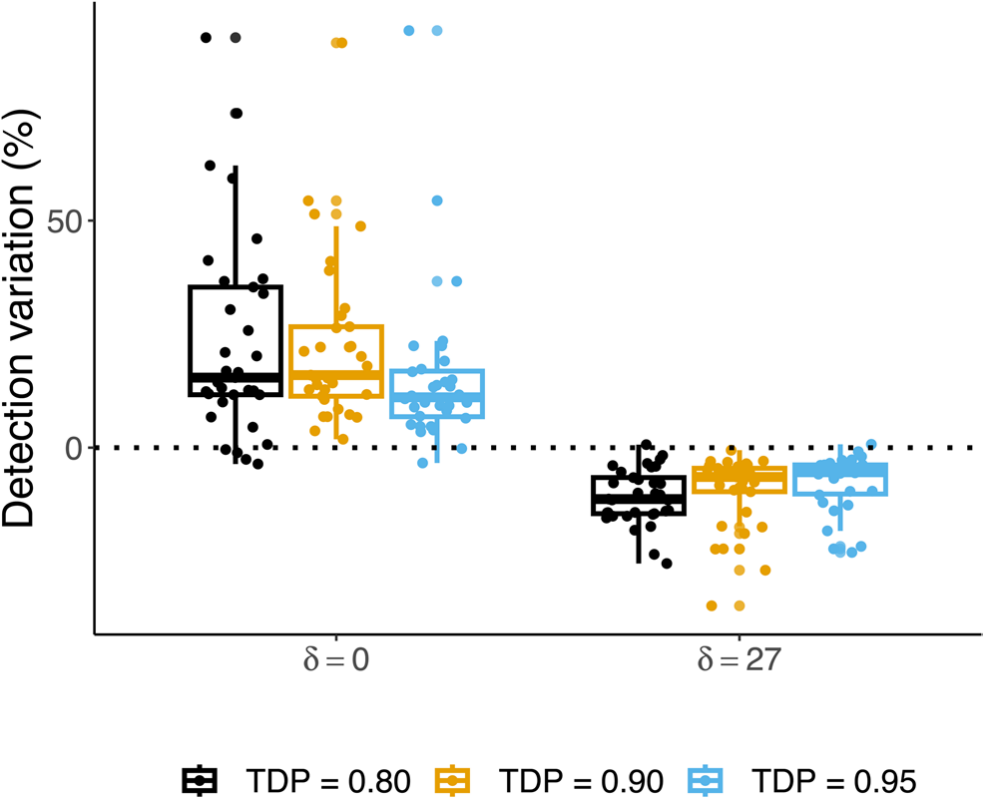
Percentage variations detected defined as$\frac{|\;\text{S}{|}_{\text{Notip}}-|\;\text{S}{|}_{\text{pARI}}}{|\;\text{S}{|}_{\text{pARI}}}$. The left side is the non-recommended setting for pARI (i.e., fixingδ=0), which we show only to reproduce the results ofBlain et al. (2022). Instead, the right side represents the results using the recommended setting for pARI as shown byAndreella et al. (2023)whenδ=27. Since the comparison is given in terms of variation as defined above, values below0indicate better performance in pARI than in Notip.

**Fig. 4.**
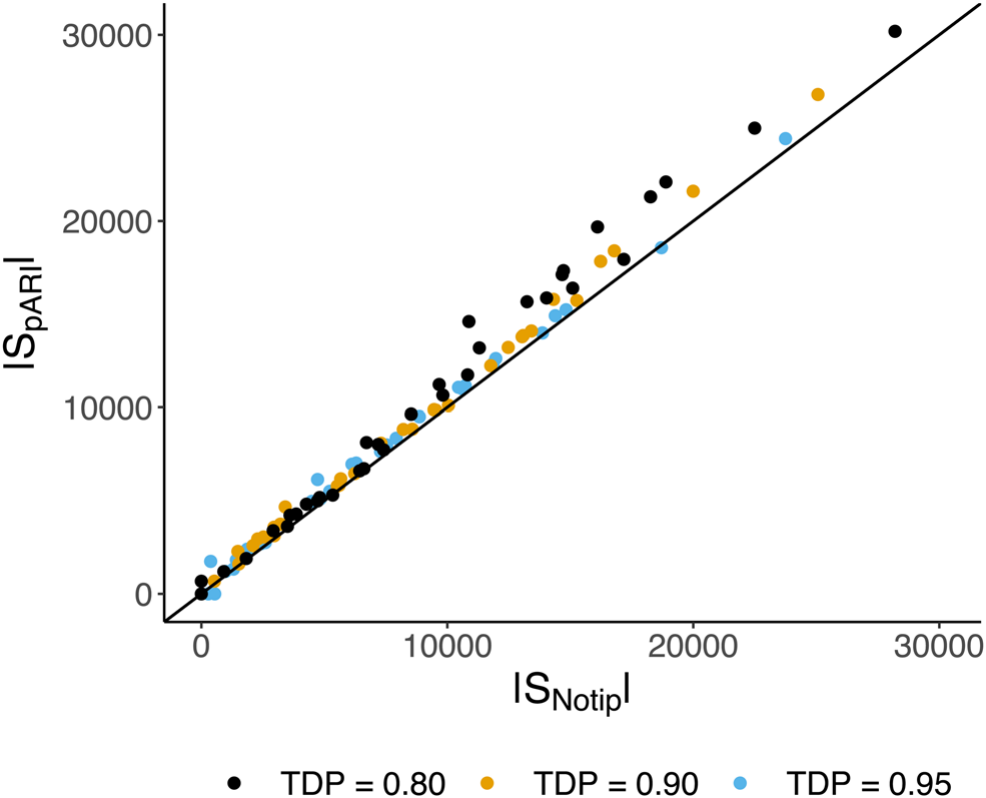
Size of the largest clusters found by pARI withδ=27($\left|{\text{S}}_{\text{pARI}}\right|$) and Notip ($\left|{\text{S}}_{\text{Notip}}\right|$) with TDP≥t∈{0.8,0.9,0.95}.

$$\frac{|S{|}_{\text{Notip}}-|S{|}_{\text{pARI}}}{|S{|}_{\text{pARI}}},$$ (7)

where|S|is the largest possible region that reaches a fixed TDP level, is analyzed. The boxplots presented inFigure 3show the distribution of this metric over36contrasts maps from Neurovault collection1952data and TDP thresholds0.8,0.9,0.95withαfixed at0.05. The results on the left-hand side ofFigure 3reproduce almost exactly the results presented inBlain et al. (2022). There are minor differences due to the use of random permutations. In addition, we noticed that the code provided byBlain et al. (2022)did not consider the mandatory inclusion of the identity transformation, which we included to get exactαcontrol (Hemerik & Goeman, 2018), even though due to the high number of permutations (i.e.,w=1,000) this makes almost no difference. The right-hand side ofFigure 3makes the same comparison but with pARI’s recommended setting ofδ=27.

Where Notip almost always outperformed pARI without the shift, we note that the reverse is true for the recommended shifted version of pARI. To investigate further,Figure 4plots the largest cluster sizes found by pARI (δ=27) against those found by Notip. Also, from this plot, we see that the size of the largest cluster found is almost always greater with pARI than with Notip, and this effect is especially pronounced when the largest cluster contains many voxels (i.e., top right part ofFigure 4).

Finally,Table 1reproduces results from Table 2 inBlain et al. (2022), to which we added results for pARI withδ=27. The contrast pair “look negative cue vs look negative rating” of the Neurovault database is analyzed. The clusters are computed by thresholding the statistical map at absolute values greater than3and keeping only clusters composed of at least150voxels (Woo et al., 2014). Again, we can note how imposingδ=27significantly increases the method’s power; pARI is, in fact, more powerful than Notip in all clusters, except the smallest one, that is, it returns greater lower bounds for the TDP.

**Table 1.** Clusters identified with threshold|z|>3*:*clusters size and TDP lower bound at risk levelα=0.05using two possible critical vectors (Notip, and Simes-based pARI withδ=27) on contrast pair “look negative cue vs look negative rating.”

		True discovery proportion		
			Simes-based pARI	
Cluster ID	Cluster size	Notip	δ=0	δ=27
1	7,695	0.26	0.23	**0.34**
2	14,877	0.45	0.32	**0.58**
3	14,445	0.50	0.37	**0.60**
4	5,238	0.29	0.24	**0.34**
5	4,563	**0.30**	**0.30**	0.29
6	12,555	0.35	0.16	**0.52**
7	6,075	0.17	0.09	**0.24**
8	25,812	0.66	0.46	**0.76**
9	6,507	0.17	0.15	**0.20**

For each cluster, the values in bold indicate the best result, that is, TDP (lower limit) higher.

We can conclude that the shifted version of Simes-based pARI performs remarkably well and, in most cases, surpasses the Notip approach, emphasizing the importance of choosing an appropriate critical vector (and shift value) for gaining power.

Please refer to the onlineSupplementary Materialsfor further analysis.

## 4 Discussion

We have seen that pARI outperformed Notip in almost all settings considered byBlain et al. (2022)when the shift parameterδof pARI was appropriately set atδ=27. This finding may seem counterintuitive since Notip uses additional information in the form of external data. It should be realized, however, that in this external data, Notip looks only marginally at the orderedp-values. The added value of this information may be limited in practice, as also illustrated by the experience (Blain et al., 2022;Meinshausen, 2006) that double dipping by reusing the data under analysis as if they were external does not break the validity of the method in practice.

Both Notip and pARI have a tuning parameter ($k_{max}$andδ, respectively). The presence of an additional parameter can be considered a drawback, especially since it has to be chosen before seeing the data. Both methods, therefore, recommend a default value ($k_{max}=1,000$andδ=27) for applications in neuroimaging. It is interesting to note that$k_{max}$andδhave complementary effects:$k_{max}<m$focuses power of Notip away from very large clusters, whileδ>0focuses power of pARI away from small ones. It could be an interesting avenue of further research to formulate an alternative method that has both a$k_{max}$and aδparameter (e.g., as considered in a different context byHemerik et al. (2019)).

It can be argued that Notip has a second tuning parameter in the choice of the external data. This can be avoided by re-use of the data under analysis, but the resulting method has no formal proof of error control. Whether data are reused or not, this additional analysis step makes the procedure more computationally expensive. For the analyses presented here (i.e., considering standard Notip and the single-step version of pARI), Notip takes approximately42minutes, while pARI takes only1minute. pARI, on the other hand, becomes computationally expensive if the step-down version is used.

Various trade-offs characterize both methods and can be seen as two out of many possible analysis choices. The comparison that we have given here shows that the choice of the family matters, but further analyses are needed to study each method’s power properties in more detail and to determine which method should be preferred in which settings. This could also help in finding even better families than those considered by Notip and pARI.

## Supplementary Material

Supplementary Material

## Data Availability

The data underlying this study are those used inBlain et al. (2022), available in the NeuroVault database athttp://neurovault.org/collections/1952. The code to preprocess the data and apply the Notip method is available at the GitHub repositoryhttps://github.com/alexblnn/Notip. The code for the pARI method is developed in the R package pARI, athttps://CRAN.R-project.org/package=pARI.
