# Supplementary material for: Selective inference for fMRI cluster-wise analysis, issues, and recommendations for critical vector selection: A comment on Blain et al
Source: Imaging Neurosci (Camb). 2024 Jun 24;2:imag-2-00198. doi: 10.1162/imag_a_00198 (PMC12272252; doi:10.1162/imag_a_00198)
Supplement: Supplementary Material [file imag_a_00198-supp.pdf]

Supplementary Materials for "Selective inference for fMRI cluster-wise analysis, issues, and recommendations for critical vector selection: A comment on Blain et al."

Angela Andreella[1], Anna Vesely[2], Wouter Weeda[3], and Jelle Goeman[4]

[1]Department of Economics, Ca' Foscari University of Venice, Venice, Italy
[2]Department of Statistical Sciences, University of Bologna, Bologna, Italy
[3]Department of Psychology, Leiden University, Leiden, The Netherlands
[4]Department of Biomedical Data Sciences, Leiden University Medical Center, Leiden, The Netherlands

The pARI approach proposed by Andreella et al. (2023) depends on the choice of the parameter δ that impacts directly the inference power. Andreella et al. (2023), after analyzing several fMRI datasets with different signal-to-noise structures, suggests the following settings: a shift of at least 1, in general, and a larger shift, set as a default at 27, if clusters composed of many voxels are of interest, as is usual in neuroimaging. In these supplementary materials, we revisit these settings to see if the choice of 27 is indeed close to optimal in the data sets considered by Blain et al. (2022).

Figure S1 shows the size of the largest cluster detected by pARI, considering several values for the shift parameter, i.e., $\delta \in \{0, 1, 3, 9, 27, 81, 243, 729, 2187\}$ analyzing the 36 pairs of contrasts from the Neurovault data, collection 1952 (Varoquaux et al., 2018) fixing TDP ≥ 0.9. The optimal δ value generally depends on the analyzed dataset. However, we can note how in the 36 pairs of contrasts analyzed, cluster size tends to increase with δ initially until it drops towards zero when δ approaches the cluster size. Since all clusters of interest are substantially larger than $\delta = 27$, this seems a reasonable choice, as found using different arguments by Andreella et al. (2023).

Table S1 extends the results presented in Table 1 by showing the lower bounds for the TDP computed by the pARI approach with $\delta \in \{0, 1, 3, 9, 27\}$. We can note that there already for $\delta = 1$ the results are competitive with Notip. Results appear to be, to an extent, fairly robust against the choice of δ, as long as a positive value is considered that is substantially smaller than the size of the smallest cluster of potential interest.

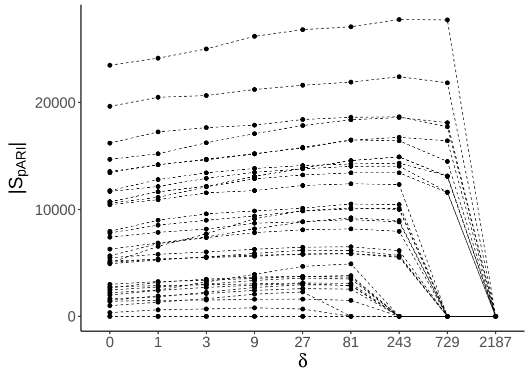

Figure S1: Largest size founded by pARI considering $\delta \in \{0, 1, 3, 9, 27, 81, 243, 729, 2187\}$ for each pair of the 36 Neurovault contrasts that reaches at least a lower bound for the TDP equals 0.9.

Cluster-ID	Cluster Size	True Discovery Proportion					
		Notip	Simes-based pARI				
			$\delta = 0$	$\delta = 1$	$\delta = 3$	$\delta = 9$	$\delta = 27$
1	7,695	0.26	0.23	0.28	0.33	**0.36**	0.34
2	14,877	0.45	0.32	0.41	0.49	0.55	**0.58**
3	14,445	0.50	0.37	0.46	0.55	0.59	**0.60**
4	5,238	0.29	0.24	0.31	0.36	**0.38**	0.34
5	4,563	0.30	0.30	0.33	**0.37**	0.36	0.29
6	12,555	0.35	0.16	0.29	0.40	0.48	**0.52**
7	6,075	0.17	0.09	0.17	0.24	**0.27**	0.24
8	25,812	0.66	0.46	0.59	0.67	0.73	**0.76**
9	6,507	0.17	0.15	0.19	0.22	**0.23**	0.20

Table S1: Clusters identified with threshold $|z| > 3$*: clusters size and TDP lower bound at risk level* $\alpha = 0.05$ *using six possible critical vectors (Notip, Simes-based pARI with* $\delta \in \{0, 1, 3, 9, 27\}$*) on contrast pair "look negative cue vs look negative rating". For each cluster, the values in bold indicate the best result, i.e., TDP (lower limit) higher.*

Finally, Figure S2 represents the largest possible region with $TDP \geq 0.9$ in the MNI space using pARI with $\delta = 27$ (top plot) and Notip (bottom plot). Here, the contrast "consonant vs baseline" versus "scramble vs baseline" of the Neurovault dataset (collection 1952) is analyzed following Blain et al. (2022)'s outline. This contrast corresponds to where the largest variation in terms of Equation (7) in the 36 contrasts analyzed was observed. Again, we can notice how the pARI approach with the recommended $\delta = 27$ returns a number of detections greater than the one produced by Notip.

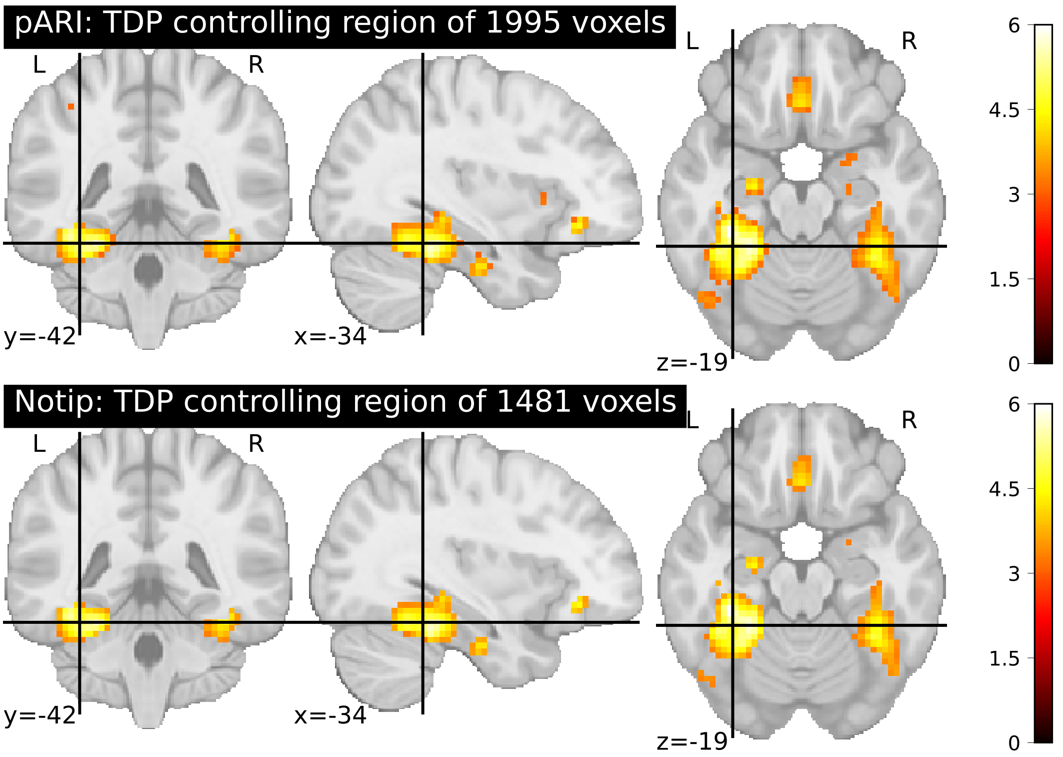

Figure S2: Statistical test map using pARI with $\delta = 27$ *(top plot) and Notip (bottom plot) considering the largest region satisfying* $TDP \geq 0.9$ *analyzing the contrast "consonant vs baseline" versus "scramble vs baseline" of the Neurovault dataset (collection* 1952*).*

References

Andreella, A., Hemerik, J., Finos, L., Weeda, W., and Goeman, J. (2023). Permutation-based true discovery proportions for functional magnetic resonance imaging cluster analysis. *Statistics in Medicine*, 42(14):2311–2340.

Blain, A., Thirion, B., and Neuvial, P. (2022). Notip: Non-parametric True Discovery Proportion control for brain imaging. *NeuroImage*, 260:119492.

Varoquaux, G., Schwartz, Y., Poldrack, R. A., Gauthier, B., Bzdok, D., Poline, J.-B., and Thirion, B. (2018). Atlases of cognition with large-scale human brain mapping. *PLoS computational biology*, 14(11):e1006565.
